# Frozen Embryo Transfer in Mildly Stimulated Cycle With Letrozole Compared to Natural Cycle in Ovulatory Women: A Large Retrospective Study

**DOI:** 10.3389/fendo.2021.677689

**Published:** 2021-09-22

**Authors:** Danjun Li, Shuzin Khor, Jialyu Huang, Qiuju Chen, Qifeng Lyu, Renfei Cai, Yanping Kuang, Xuefeng Lu

**Affiliations:** Department of Assisted Reproduction, Shanghai Ninth People’s Hospital, Shanghai Jiaotong University School of Medicine, Shanghai, China

**Keywords:** letrozole, endometrial preparation, frozen embryo transfer (FET), clinical pregnancy, ovulatory (menstrual) cycle

## Abstract

**Objective:**

To evaluate the clinical effect of mild stimulation with letrozole on pregnancy outcomes in ovulatory women undergoing frozen embryo transfer (FET) compared to natural cycle.

**Design:**

Retrospective observational study.

**Setting:**

Tertiary care academic medical center.

**Population:**

A total of 6,874 infertile women with regular menstrual cycles (21-35 days) met the criteria for this study in the period from 2013 to 2020.

**Methods:**

All patients who were prepared for and underwent FET were divided into two groups: a modified natural cycle (NC) group (n=3,958) and a letrozole cycle group (n=2,916).

**Main Outcome Measures:**

The primary outcome of the study was clinical pregnancy rate. Secondary outcome measures were endometrial thickness, rates of implantation, positive HCG test, live birth, early miscarriage and ectopic pregnancy.

**Results:**

The clinical pregnancy rate was not statistically different between the modified NC-FET group and the letrozole-FFT group before (crude OR 0.99, 95% CI 0.90-1.09, P=0.902>0.05) and after propensity score matching (PSM) (crude OR 1.01, 95% CI 0.91-1.12, P=0.870>0.05). After multivariable logistic regression analysis, the clinical pregnancy rate remained insignificant before (adjusted OR 1.00, 95% CI 0.91-1.10, P=0.979>0.05) and after matching (adjusted OR 1.00, 95% CI 0.89-1.11, P=0.936>0.05), respectively. Similarly, in the crude and adjusted analysis, the positive HCG test, implantation, live birth and early miscarriage rates were also comparable in the letrozole-FFT group and modified NC-FET group before and after matching. Furthermore, the endometrial thickness of letrozole-FFT group was similar to that of modified NC-FET group with adjusted analysis.

**Conclusion:**

Our observation suggests that mild stimulation with letrozole could produce similar pregnancy outcomes in ovulatory patients who undergo FET when compared with a natural cycle.

## Introduction

Implantation is a critical step of successful *in vitro* fertilization (IVF) therapy for infertile couples. With the contribution of several factors, including advent of vitrification techniques (1), improvement in culture media and selection of cryopreservation strategy, frozen embryo transfer (FET) is now widespread (2, 3).

To optimize pregnancy rates, various regimens and cycles have been used to prepare the endometrium during FET (4–6). Frozen-thawed embryos can be transferred to the uterus during the natural cycle, a mildly ovarian stimulated cycle, or an artificial cycle in women with regular menstrual cycles. Nonetheless, based on previous studies comparing different cycle regiments for FET, there is no evidence that one cycle regimen is superior to another cycle regimen (7, 8).

Letrozole, a third-generation aromatase inhibitor, does not antagonize estrogen receptors and maintains the normal central feedback required to facilitate normal follicular growth, selection of dominant follicles and ovulation without negatively affecting the endometrium (9). Recently, it is noticed whether letrozole use could benefit pregnancy outcomes when compared with natural or hormone replacement therapy (HRT) cycles. Tatsumi et al. found that use of letrozole in FET was associated with significantly higher rates of clinical pregnancy, clinical pregnancy with fetal heart beat and live birth, as well as a significantly lower rate of miscarriage compared to natural or HRT cycles in women undergoing either early cleavage or blastocyst embryo transfer (10). However, this study lacked precise information on patients, such as anovulatory/dysovulatory (i.e., polycystic ovary syndrome), and embryo quality, which might have biased the results. Recently, mild stimulation with letrozole was demonstrated to be associated with a significantly higher maximal endometrial thickness and higher rates of clinical pregnancy, ongoing pregnancy, and implantation compared to using hormonal manipulation or HMG stimulation in endometrial preparation for FET in polycystic ovary syndrome (PCOS) patients (11). Actually, our recent observational study also revealed that mild stimulation with letrozole for endometrial preparation was associated with a higher live birth rate compared to an HRT group in patients with PCOS undergoing FET (12). Further molecular studies suggested that mild stimulation with letrozole might improve endometrial receptivity and embryo implantation. Letrozole use for ovulation in PCOS patients increase a number of markers of endometrial receptivity, such as LIF, DKK1, LIFR, and FGF-22, which might have a positive effect on embryo implantation (13). Miller P.B. et al. recently showed that letrozole increased endometrial ανβ3 integrin expression and improves implantation and pregnancy rates (14).

However, so far, whether the use of mild stimulation with letrozole during FET cycles (L-FET) benefits pregnancy outcomes in patients with regular ovulatory cycles compared to natural cycle prior to FET (NC-FET), which is the most popular strategy for endometrium preparation in ovulatory women, remains largely unknown. In the current large retrospective study, we aimed to compare reproductive outcomes in women with regular menstrual cycles who underwent modified NC-FET or L-FET cycles.

## Materials and Methods

### Study Design and Participants

This retrospective study was performed at the Department of Assisted Reproduction of the Ninth People’s Hospital of Shanghai JiaoTong University School of Medicine during the period from November 2013 to December 2020. The following were inclusion criteria: (1) infertile women with regular menstrual cycles (21-35 days); (2) first FET cycle; (3) females aged <40 years at oocyte retrieval and embryo transfer; (4) transferred embryos at cleavage stage. The following were exclusion criteria: women diagnosed with PCOS according to the 2003 Rotterdam ESHRE/ASRM-Sponsored PCOS Consensus Workshop (15). All patients full-filling the inclusion and exclusion criteria were invariably included in our study. This study protocol was approved by the institutional ethics committee of the ninth hospital. The flow diagram of cohort selection is shown in **Supplemental Figure 1**.

### Endometrial Preparation Before Embryo Transfer

All embryos were frozen on day 3 of preimplantation development in a previous treatment cycle. The choice of either modified NC or mild stimulation with letrozole was based on physician preference. In the letrozole group, patients were orally administered letrozole (Femara; Norvatis, East Hanover, NJ, USA) from day 3 of the menstrual cycle (MC) for 3 days, at a daily dose of 2.5 mg, and ultrasound monitoring and serum hormone analysis were performed from MC10. Should it be a dominant follicle < 14 mm on day 10, intramuscular injection of 150 IU HMG every other day (Fengyuan Pharmaceutical Co. Anhui, China) was added starting on MC10. In the modified NC group, patients were monitored by serial transvaginal scanning and hormone assays starting from MC10. Follicle monitoring, hormone measurement, and the way of hCG triggering were provided as the letrozole group.

In both groups, when the diameter of dominant follicle was >17 mm and endometrial thickness was ≥ 7 mm, with E_2_ levels preferably >150 pg/mL, ovulation was triggered with hCG, whose triggering time was dependent on the occurrence of LH surge or serum level of P. If a serum LH surge was detected (LH≥20 IU/L) or serum P level ≥ 1 ng/ml, a bolus of hCG (5,000 IU; Lizhu Pharmaceutical Trading Co. Zhuhai, China) was injected in the same afternoon. Patients began oral administration of 40 mg dydrogesterone (Abbott Healthcare Products, B.V., Weesp, Netherlands) and vaginal administration of 400 mg micronized progesterone (Utrogestan, Laboratoires Besins-Iscovesco, Paris, France) daily for 2 days after ovulatory trigger and embryo transfer (ET) was performed 2 days later under abdominal ultrasound guidance. In the absence of an LH surge (LH <20 IU/L) and serum P level < 1 ng/ml, hCG was injected at 9:00 p.m. Exogenous progesterone supplementation was initiated for 3 days after ovulatory trigger, and ET was performed 2 days later. If endometrial thickness did not reach 7 mm, the cycle was cancelled. Progesterone supplementation continued until 12 weeks of pregnancy. The schematic diagram for embryo transfer is shown in **Supplemental Figure 2**.

### Embryo Vitrification, Thawing, and Transfer

The cleavage-stage embryos (Day 3) were graded according to the Cummins’s criteria (16). The high quality embryos (such as grade 1 and grade 2) were selected and frozen by vitrification after oocyte retrieval. The vitrification procedure for freezing day 3 embryo was performed using the Cryotop carrier system (Kitazato Biopharma Co.). Briefly, embryos were put into the pre-warmed Equilibration Solution (ES) for 5 minutes and successively were transferred into the pre-warmed Vitrification Solution (VS) for no more than 1 minutes. Then, *via* the Cryotop carrier, the embryos were plunged into the liquid nitrogen for storage. For thawing, embryos were transferred from liquid nitrogen into warming solution at the temperature of 37°C for 1 minutes, and afterwards transferred into dilution solution in proper order. Subsequently, the thawed embryos were put into equilibrated culture droplet and further cultured for 2-3 hours until the transfer. All Day 3 embryos were thawed on the day of transfer and no more than two embryos were transferred.

### Outcome Measures

The primary outcome of this study was the presence of clinical pregnancy per embryo transfer. The secondary outcome measures included endometrial thickness, implantation rate, positive hCG pregnancy rate, live birth rate, early miscarriage rate and ectopic pregnancy rate.

Clinical pregnancy was defined as the presence of at least one gestational sac on ultrasound after FET at 5 weeks of gestation. Live birth was defined as live born babies ≥28 gestational weeks. Implantation rate was defined as the total number of gestational sacs inside the uterine cavity observed by ultrasound divided by the total number of transferred embryos. Early miscarriage was defined as a loss of clinical pregnancy prior to 12 gestational weeks. Ectopic pregnancy was defined as a gestational sac outside of the uterine cavity during ultrasound examination. Intrauterine and ectopic pregnancy was defined as simultaneous existence of gestational sacs inside and outside the uterine cavity observed by ultrasound.

### Statistical Analysis

All statistical analyses were performed using the Statistical Package for Social Sciences (version 25.0; SPSS Inc., USA) and R statistical programming language (version 4.1.0; R Foundation for Statistical Computing, Austria). A P value <0.05 was considered statistically significant. For continuous variables, the normality was tested by the Kolmogorov-Smirnov test as well as histograms and Q-Q plots. If data were normally distributed, they were expressed as the mean ± standard deviation (SD), otherwise, they were expressed as median (25^th^ percentile-75^th^ percentile); while for categorical variables, they were expressed as number (percentage). Continuous variables were compared *via* Student’s t-test (normal distribution) or Mann-Whitney U test (no normal distribution), while categorical variables were compared *via* Chi-squared test.

To balance baseline characteristics between two groups, a one to one propensity score matching (PSM) model was established using the nearest-neighbor matching algorithm. Confounding factors, such as age, body mass index (BMI), duration of infertility, cause of infertility, duration of cryopreservation, number of transferrable embryos, embryo quality at transfer, endometrium thickness, number of previous fresh embryo transfer attempt and hormones on triggering day were chosen for matching. The balance between two groups after matching was evaluated by the standardized mean difference (<0.1).

The association between the type of endometrial preparation and pregnancy outcome was further evaluated before and after PSM using both univariable and multivariable logistic regression analysis. All the aforementioned confounders for matching were introduced into the regression equation for adjustment by the enter method. We calculated crude and adjusted odds ratios (ORs) with corresponding 95% confidence intervals (CIs).

## Results

### Patient and Cycle Characteristics

Between November 2013 and December 2020, a total of 6,874 women with regular menstruation (21-35 days) who were enrolled in this study were stratified into two cohorts: modified natural cycle FET (NC-FET) and letrozole cycle FET (L-FET). Among them, 3,958 women underwent modified NC, while 2,916 women underwent letrozole cycle (**Table 1**). Of the letrozole group, 1888 women received HMG therapy. And 355 women undergoing first FET cycles in 2020 were lack of live birth data, so the remaining 6419 women were included in analysis for live birth rate.

**Table 1:** Patient demographics and cycle characteristics.

	Before matching			After matching		
	mNC-FET (*n* = 3958)	L-FET (*n* = 2916)	*P*-value	mNC-FET (*n* = 2692)	L-FET (*n* = 2692)	*P*-value
Age (years)	31.36 ± 3.37	30.93 ± 3.56	<0.001	31.11 ± 3.38	31.05 ± 3.52	0.540
Body mass index (kg/m^2^)	21.25 ± 2.76	21.70 ± 3.11	<0.001	21.52 ± 2.79	21.46 ± 2.83	0.402
Duration of infertility (years)	3 (1-4)	3 (1-4)	0.870	2 (1-4)	3 (1-4)	0.543
Cause of infertility, *n* (%)			0.091			0.558
Male factor	412 (10.4)	334 (11.5)		296 (11.0)	306 (11.4)	
Tubal factor	1472 (37.2)	1105 (37.9)		994 (36.9)	1025 (38.1)	
Endometriosis	75 (1.9)	42 (1.4)		50 (1.9)	40 (1.5)	
Unexplained	112 (2.8)	103 (3.5)		82 (3.0)	92 (3.4)	
Combined	1887 (47.7)	1332 (45.7)		1270 (47.2)	1229 (45.7)	
Hormones on triggering day, *n* (%)						
E2 ≤ 150 pg/ml	813 (20.5)	564 (19.3)	0.220	540 (20.1)	531 (19.7)	0.759
P ≥ 1 ng/ml	704 (17.8)	576 (19.8)	0.038	518 (19.2)	507 (18.8)	0.703
LH ≥ 20 IU/ml	1725 (43.6)	1113 (38.2)	<0.001	1043 (38.7)	1060 (39.4)	0.635
Duration of cryopreservation (years)	0.30 ± 0.29	0.27 ± 0.23	<0.001	0.28 ± 0.18	0.27 ± 0.18	0.868
No. of embryos transferred, *n* (%)	2 (2-2)	2 (2-2)	<0.001	2 (2-2)	2 (2-2)	0.530
Single	545 (13.8)	539 (18.5)	<0.001	446 (16.6)	429 (15.9)	0.530
Double	3413 (86.2)	2377 (81.5)		2246 (83.4)	2263 (84.1)	
Post-thaw embryo survival rate	7419/7431 (99.8)	5308/5311 (99.9)	0.116	4968/4978 (99.8)	4968/4972 (99.9)	0.179
Embryo quality at transfer of top embryo transferred, *n* (%)			0.001			0.054
1	363 (9.2)	207 (7.1)		213 (7.9)	201 (7.5)	
2	3518 (88.9)	2672 (91.6)		2422 (90.0)	2456 (91.2)	
3	77 (1.9)	37 (1.3)		57 (2.1)	35 (1.3)	
No. of previous fresh embryo transfer attempt, *n* (%)			0.068			0.747
0	3749 (94.7)	2790 (95.7)		2563 (95.2)	2568 (95.4)	
1	209 (5.3)	126 (4.3)		129 (4.8)	124 (4.6)	
Endometrial thickness (mm)	10.69 ± 2.18	10.84 ± 2.28	0.012	10.77 ± 2.18	10.78 ± 2.23	0.867

Data are given as mean ± SD (normal distribution) and median (25^th^ percentile-75^th^ percentile) (no normal distribution) for continuous variables and n (%) for dichotomous variables.

All P values were assessed with the use of x^2^ or Fisher’s exact test (dichotomous variables) and t test or Mann-Whitney U test (continuous variables).

mNC, modified natural cycle; L, letrozole; FET, frozen-thawed embryo transfer; IVF, in vitro fertilization; ICSI, intracytoplasmic sperm injection.

The baseline characteristics before (left column) and after PSM (right column) were shown in **Table 1**. Before matching, a comparison of patient demographics indicated that the age of women in the L-FET group was slightly younger than women in the modified NC-FET group (30.93 ± 3.56 *vs*. 31.36 ± 3.37, P<0.001), while the BMI of women in the L-FET group was slightly higher than women in the modified NC-FET group (21.70 ± 3.11 *vs*. 21.25 ± 2.76, p<0.001) (**Table 1**). Moreover, the duration of embryo cryopreservation in the modified NC-FET group was slightly longer than that of embryo cryopreservation in the L-FET group (0.30 ± 0.29 *vs*. 0.27 ± 0.23, P<0.001) (**Table 1**). No significant difference was observed between the two treatment groups with respect to cause of infertility or duration of infertility.

Then for cycle characteristics, the proportion of E2 deficiency on triggering day, the embryo survival rate after thawing and the number of previous fresh ET cycle were similar in both groups presented in **Table 1**. The proportion of P rise and LH rise in NC-FET group and L-FET group were 17.8% *vs*. 19.8% (P=0.038<0.05) and 43.6% *vs*. 38.2% (P<0.001), respectively (**Table 1**). Significantly fewer proportion of double embryos and consequently fewer number of embryos were transferred in L-FET group compared to modified NC-FET group (81.5% *vs*. 86.2%, P<0.001). However, significantly more high-quality embryos (grade1 and grade 2) were transferred in L-FET group compared to modified NC-FET group (98.7% *vs*. 98.1%, P=0.001<0.05) (**Table 1**). Furthermore, the endometrial thickness was significantly greater in L-FET group than in modified NC-FET group (10.84 ± 2.28 *vs*. 10.69 ± 2.18, P=0.012<0.05) (**Table 1**). Given that HMG could stimulate the growth of endometrium, we further divided L-FET group into letrozole group and letrozole +HMG group and made a comparison of endometrial thickness among three groups (**Supplemental Table 1**). It was observed that there was no significant difference among three groups.

After matching, all baseline characteristics of 2692 women each group were similarly adjusted, and there was no significant difference observed between the two treatment groups regarding all confounders (**Table 1**).

### Reproductive Outcomes

In the crude analysis, the clinical pregnancy rate was not statistically different for women between the L-FET group and the modified NC-FET group (53.9% *vs*. 54.1%, P=0.902>0.05). Women in the letrozole cycle group exhibited similar positive hCG test rate (57.4% *vs*. 58.2%, p=0.500>0.05) and implantation rate (0.37 ± 0.40 *vs*. 0.37 ± 0.39, P=0.951>0.05) compared to women in the modified NC-FET group (**Table 2**). Likewise, the live birth rate per transfer cycle was comparable between the L-FET group and the modified NC-FET group (46.6% *vs*. 46.5%, p=0.940>0.05). Furthermore, no significant between-group differences were observed in the early miscarriage rate, ectopic pregnancy rate, or intrauterine/ectopic pregnancy rate (**Table 2**). Moreover, we further analyzed that no evident differences existed for clinical pregnancy rate and live birth rate per single or double embryo transfer (**Table 2**).

**Table 2:** Pregnancy outcomes between the natural and letrozole groups.

	Before Matching			After matching		
	mNC-FET (*n* = 3958)	L-FET (*n* = 2916)	*P*-value	mNC-FET (*n* = 2692)	L-FET (*n* = 2692)	*P*-value
Positive HCG test, *n* (%)	2303 (58.2)	1673 (57.4)	0.500	1570 (58.3)	1560 (57.9)	0.782
Clinical pregnancy, *n/N* (%)	2141 (54.1)	1573 (53.9)	0.902	1461 (54.3)	1467 (54.5)	0.870
SET	201/545 (36.9)	211/539 (39.1)	0.442	171 (38.3)	170 (39.6)	0.697
DET	1940/3413 (56.8)	1362/2377 (57.3)	0.729	1290 (57.4)	1297 (57.3)	0.934
Live birth, *n/N* (%) *	1769/3806 (46.5)	1217/2613 (46.6)	0.940	1204/2578 (46.7)	1148/2443 (47.0)	0.838
SET	128/454 (28.2)	107/358 (29.9)	0.597	109 (29.4)	90 (30.4)	0.774
DET	1641/3352 (49.0)	1110/2255 (49.2)	0.844	1095 (49.6)	1058 (49.3)	0.824
Implantation rate (%)	0.37 ± 0.39	0.37 ± 0.40	0.951	0.37 ± 0.39	0.37 ± 0.39	0.917
Early miscarriage, *n/N* (%)	131/2141 (6.1)	88/1573 (5.6)	0.503	92/1461 (6.3)	77/1467 (5.2)	0.224
Ectopic pregnancy, *n/N* (%)	44/2141 (2.1)	45/1573 (2.9)	0.113	34/1461 (2.3)	42/1467 (2.9)	0.362
Intrauterine and ectopic pregnancy, *n/N* (%)	8/2141 (0.4)	6/1573 (0.4)	0.970	8/1461 (0.5)	6/1467 (0.4)	0.587

Data are presented as mean ± SD for continuous variables and n (%) for dichotomous variables. All P values were assessed with the use of x^2^ test (dichotomous variables) and t test (continuous variables). * The rate of live birth was evaluated between 2013 and 2019.

mNC, modified natural cycle; L, letrozole; FET, frozen-thawed embryo transfer; SET, Single embryo transfer; DET, Double embryo transfer.

The details of neonatal outcomes are provided separately for singletons and twins. Among neonates, no notable differences were observed in the characteristics, e.g., sex of neonate, rates of preterm delivery and low birth weight. Moreover, the rate of major congenital anomalies in the L-FET group was comparable to the rate in the NC-FET group (**Table 3**).

**Table 3:** Neonatal outcome of live born infants between the natural and letrozole groups in the period from 2013 to 2019.

	Before matching			After matching		
	NC-FET (*n* = 1769)	L-FET (*n* = 1217)	*P*-value	NC-FET (*n* = 1204)	L-FET (*n* = 1148)	*P*-value
**Singleton**						
No. of neonate	1277	898		890	847	
Sex of neonate			0.455			0.311
Male	660 (51.7)	456 (50.8)		460 (51.7)	427 (50.0)	
Female	617 (48.3)	441 (49.1)		430 (48.3)	418 (49.4)	
Unknown	0 (0)	1 (0.1)		0 (0)	2 (0.2)	
Low birth weight (<2500 g), *n* (%)	39 (3.1)	35 (3.9)	0.285	24 (2.7)	34 (4.0)	0.127
Preterm birth (<37 weeks), *n* (%)	54 (4.2)	40 (4.5)	0.799	35 (3.9)	40 (4.7)	0.418
Major congenital anomalies, *n* (%)	18 (1.4)	17 (1.9)	0.378	13 (1.5)	15 (1.8)	0.608
**Twins**						
No. of children	984	638		628	602	
Sex of neonate			0.238			0.162
Male	498 (50.6)	342 (53.6)		314 (50.0)	325 (54.0)	
Female	486 (49.4)	296 (46.4)		314 (50.0)	277 (46.0)	
Low birth weight (<2500 g), *n* (%)	356 (36.2)	236 (37.0)	0.740	233 (37.1)	226 (37.5)	0.873
Preterm birth (<37 weeks), *n* (%)	304 (30.9)	226 (35.4)	0.057	210 (33.4)	214 (35.5)	0.437
Major congenital anomalies, *n* (%)	15 (1.5)	14 (2.2)	0.320	20 (3.2)	14 (2.3)	0.358

Data are given as mean ± SD for continuous variables and n (%) for dichotomous variables.

All P values were assessed with the use of x^2^ or Fisher’s exact test.

NC, natural cycle; L, letrozole; FET, frozen-thawed embryo transfer.

**Table 4** shows crude ORs and adjusted ORs. After correcting for confounding factors, the clinical pregnancy rate stratified by the number of embryos transferred remained not statistically different in women undergoing L-FET and NC-FET (adjusted OR 1.09, 95% CI 0.84-1.41; adjusted OR 0.99, 95% CI 0.89-1.10). Similarly, the same result was observed regarding live birth rate stratified by the number of embryos transferred (adjusted OR 1.12, 95% CI 0.82-1.53; adjusted OR 0.97, 95% CI 0.87-1.08). The prevalence of positive HCG test was not statistically different between two groups (adjusted OR 0.97, 95% CI 0.88-1.07) (**Table 4**). The adjusted OR of the early miscarriage rate was 0.90 (95% CI 0.68-1.19), which was still not statistically significant (**Table 4**). Consistently, re-analysis after PSM showed that pregnancy outcomes remained insignificant (**Table 4**).

**Table 4:** Crude and adjusted odds ratios (ORs) of pregnancy outcomes between the natural and letrozole groups.

	Before matching				After matching			
	Crude OR (95% CI)	*P-*value	Adjusted OR (95% CI)	*P*-value	Crude OR (95% CI)	*P-*value	Adjusted OR (95% CI)	*P-*value
Positive HCG test rate	0.97 (0.88-1.07)	0.500	0.97 (0.88-1.07)	0.577	0.99 (0.88-1.10)	0.782	0.97 (0.87-1.08)	0.594
Clinical pregnancy rate	0.99 (0.90-1.09)	0.902	1.00 (0.91-1.10)	0.979	1.01 (0.91-1.12)	0.870	1.00 (0.89-1.11)	0.936
SET	1.10 (0.86-1.41)	0.442	1.09 (0.84-1.41)	0.517	1.06 (0.80-1.39)	0.697	1.08 (0.81-1.43)	0.597
DET	1.02 (0.92-1.13)	0.729	0.99 (0.89-1.10)	0.807	1.00 (0.88-1.12)	0.934	0.99 (0.87-1.11)	0.801
Early miscarriage	0.91 (0.69-1.20)	0.503	0.90 (0.68-1.19)	0.451	0.82 (0.60-1.13)	0.225	0.83 (0.60-1.13)	0.230
Live birth rate*	1.00 (0.91-1.11)	0.940	0.98 (0.89-1.09)	0.740	1.01 (0.91-1.13)	0.838	0.98 (0.88-1.10)	0.732
SET	1.09 (0.80-1.47)	0.597	1.12 (0.82-1.53)	0.485	1.05 (0.75-1.47)	0.774	1.11 (0.79-1.56)	0.559
DET	1.01 (0.91-1.13)	0.844	0.97 (0.87-1.08)	0.605	0.99 (0.88-1.11)	0.824	0.97 (0.86-1.09)	0.627

Analyses were adjusted for age, body mass index, duration of infertility, cause of infertility, hormones on triggering day, duration of cryopreservation, number of transferrable embryos, embryo quality at transfer, number of previous fresh embryo transfer attempt, endometrium thickness. *The rate of live birth was evaluated between 2013 and 2019.

OR, odds ratio; CI, confidence interval; SET, Single embryo transfer; DET, Double embryo transfer.

Crude and adjusted ORs for neonatal outcomes are shown in **Supplemental Table 2**. The adjusted ORs of the L-FET group for both singletons and twins were similar to those for the NC-FET group (**Supplemental Table 2**). Similarly, the neonatal outcomes were demonstrated invariably after re-analysis using PSM.

## Discussion

Previous studies have argued that whether letrozole could improve endometrial receptivity and benefit pregnancy outcomes. At present, it is unclear whether mild stimulation with letrozole results in higher implantation rates and clinical pregnancy rates compared to natural cycle in ovulatory women. To our knowledge, our study is the first large retrospective study to illustrate detailed information on patient and cycle characteristics with the comparison of L-FET and NC-FET in ovulatory women. The findings of this study demonstrated that mild stimulation with letrozole is not associated with higher chances of clinical pregnancy and implantation compared to the NC group in ovulatory women.

Many studies have examined the efficacy of mild stimulation with letrozole for endometrial preparation before FET. However, a great majority of studies have focused on PCOS patients and confirmed the effectiveness of letrozole. As yet no study has compared letrozole mild stimulation to the natural cycle, which is the most popular protocol for endometrium preparation in ovulatory women. Previous study has demonstrated that integrin expression, a marker of endometrial receptivity, was of vital importance for the success of IVF treatment and its deficiency was associated with IVF failure (17). Afterwards, Ganesh et al. has investigated that stimulated cycle with letrozole could improve the integrin expression, namely endometrial receptivity, as compared to NC (18). Miller et al. also examined that integrin expression were increased and pregnancy outcomes were improved with the use of letrozole and gonadotropin (14).

Our present study aimed to analyze the clinical pregnancy outcomes between letrozole cycle and natural cycle groups by including patients with normal menstrual cycles, taking important confounders into consideration as much as possible. It has been reported that letrozole use inhibited the aromatase enzyme, resulting in a blockade of androgen conversion into estrogen and a subsequent increase in androgens along with a decrease in estrogens (19). Low estrogen concentration upregulates estrogen receptors and increases sensitivity to subsequent estrogen increases, which might result in faster proliferation of the endometrial epithelium and favor embryo implantation (20). In our current study, it’s maybe because of large sample size, the L-FET cycles group has significantly greater endometrial thickness in contrast with NC-FET group. To rule out the potential effect of HMG, we have made a further grouping, dividing L-FET group into letrozole group and letrozole + HMG group, and made a comparison of endometrial thickness categorized according to the cut-offs by the 10^th^ (8.2mm), 50^th^ (10.5mm) and 90^th^ (13.7mm) percentile of the whole endometrial thickness. And the categorization was consistent with previous studies (21, 22). It was demonstrated that as a matter of fact the endometrial thickness was comparable among only letrozole group, letrozole + HMG group and NC group, indicating that letrozole could not promote endometrial proliferation and favor embryo implantation. Furthermore, comparable pregnancy outcomes were observed between letrozole cycle group and NC group, which suggests that letrozole mildly stimulation might produce pregnancy outcomes in accordance with the normal spontaneous ovulation cycle.

There was no significant difference in the neonatal outcome of live born infants between L-FET and NC-FET groups. This result is consistent with a previous study on offspring. It has been verified that letrozole use yields similar outcomes to the natural cycles with respect to neonatal outcomes in Tatsumi’s study in Japan (10). A previous study in humans demonstrated that there were no major or minor congenital malformations linked to letrozole use due to its short half-life (23).

We acknowledge that our study does have shortcomings. First, as our investigation was a retrospective observational study, patients were assigned to different regiments based on clinical practice, which could easily lead to selection bias; therefore, a prospective cohort study should be further conducted. Furthermore, although we used multivariable logistic regression to control for confounders between the two groups, we could not control for all confounders. Then, our clinical data were also all from a single reproductive center; therefore, further studies on this subject are needed to gain the support of multiple reproductive centers. Finally, the cycle cancellation rate was not analyzed in this study.

With respect to study strengths, the highlight of the present study is based on its large sample size to make our results more reliable. However, due to large sample size of the study, we could see that there were a lot of statistically significant differences, which were actually small, concerning patient demographics and cycle characteristics. Therefore, the highly statistical difference may be contributed to large sample size, not meaning clinical relevance. Thus, we have applicated the PSM to make the baseline characteristics similar. By using multivariable regression analysis controlling for confounding variables and PSM for re-analysis, the robustness of the results was confirmed. In addition, our study comes from actual clinical data instead of a clinical trial, which avoids strict screening criteria that could limit the extrapolation of results.

In conclusion, our study confirms that, for women with regular menstrual cycles, L-FET could produce comparable pregnancy outcomes in contrast to NC-FET. The mild stimulation with letrozole demands quite more effort and costs while natural cycle regimen is easy and inexpensive. Considering these, we recommend the natural cycle for endometrial preparation prior to embryo transfer as the treatment of choice in women with normal ovulation. In the future, a multi-center randomized controlled trial (RCT) is still needed to test and verify the conclusions of this study.

## Data Availability Statement

The original contributions presented in the study are included in the article/**Supplementary Material**. Further inquiries can be directed to the corresponding authors.

## Ethics Statement

The studies involving human participants were reviewed and approved by the institutional ethics committee of the ninth hospital. The patients/participants provided their written informed consent to participate in this study.

## Author Contributions

XL and YK designed the study. DL and SK collected the data, performed the statistical analysis and drafted the manuscript. JH, QC, QL, and RC helped making interpretation for the data. All authors contributed to the article and approved the submitted version.

## Funding

This research was supported by grants from National Natural Science Foundation of China (no.81873856, 81771533), the “Two-hundred Talent” (20191814), Shanghai Health and Family Planning Commission (201940287) and National Key R&D Program of China (2018YFC1003000).

## Conflict of Interest

The authors declare that the research was conducted in the absence of any commercial or financial relationships that could be construed as a potential conflict of interest.

## Publisher’s Note

All claims expressed in this article are solely those of the authors and do not necessarily represent those of their affiliated organizations, or those of the publisher, the editors and the reviewers. Any product that may be evaluated in this article, or claim that may be made by its manufacturer, is not guaranteed or endorsed by the publisher.
