# Supplementary material for: Frozen Embryo Transfer in Mildly Stimulated Cycle With Letrozole Compared to Natural Cycle in Ovulatory Women: A Large Retrospective Study
Source: Front Endocrinol (Lausanne). 2021 Sep 22;12:677689. doi: 10.3389/fendo.2021.677689 (PMC8493067; doi:10.3389/fendo.2021.677689)
Supplement: Supplementary file 1 [file DataSheet_1.doc]

**Supplemental Files**

**Supplemental Figure 1** Flow diagram of cohort screening in the study


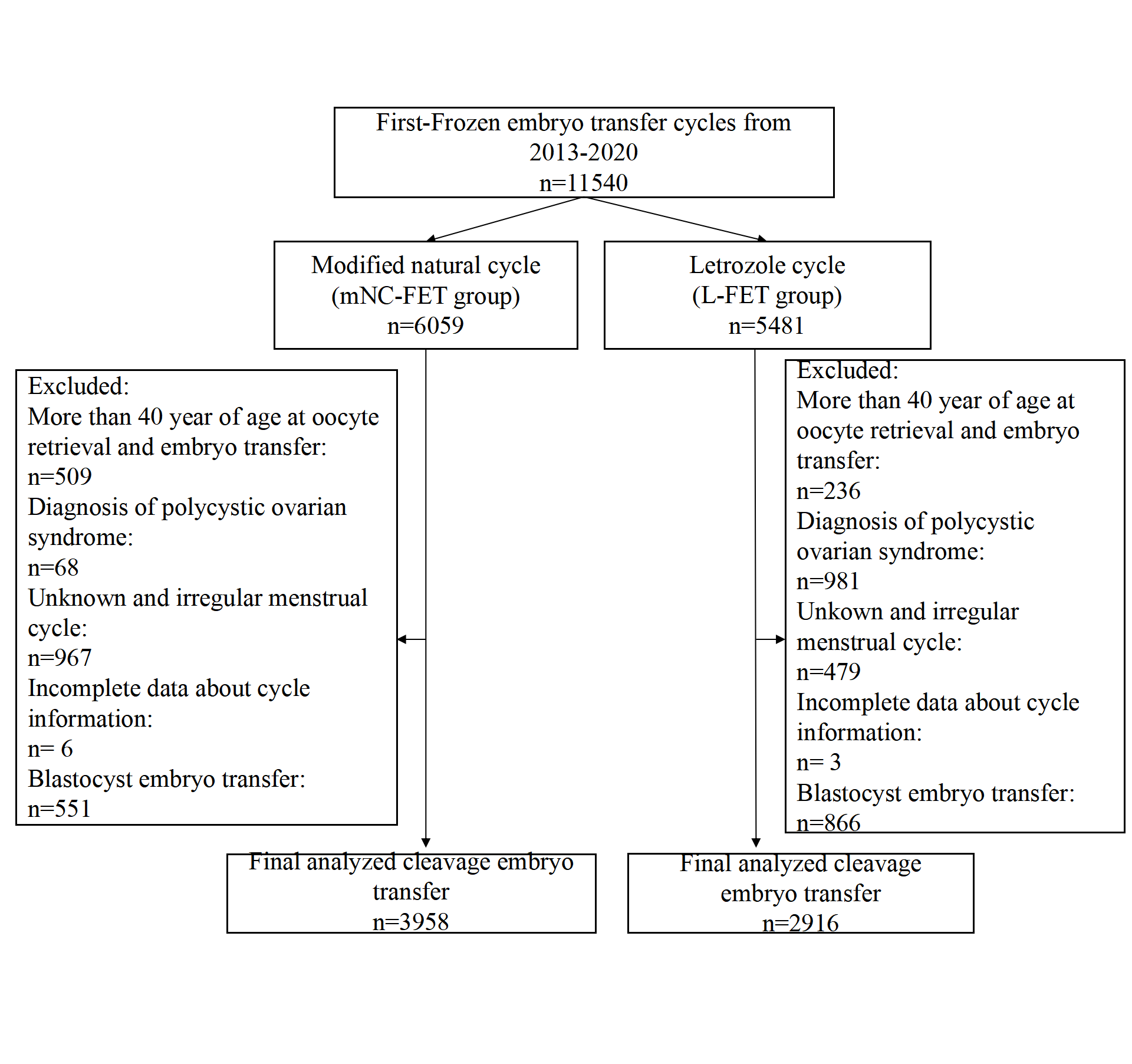


**Supplemental figure 2** The schematic diagram for embryo transfer.

**
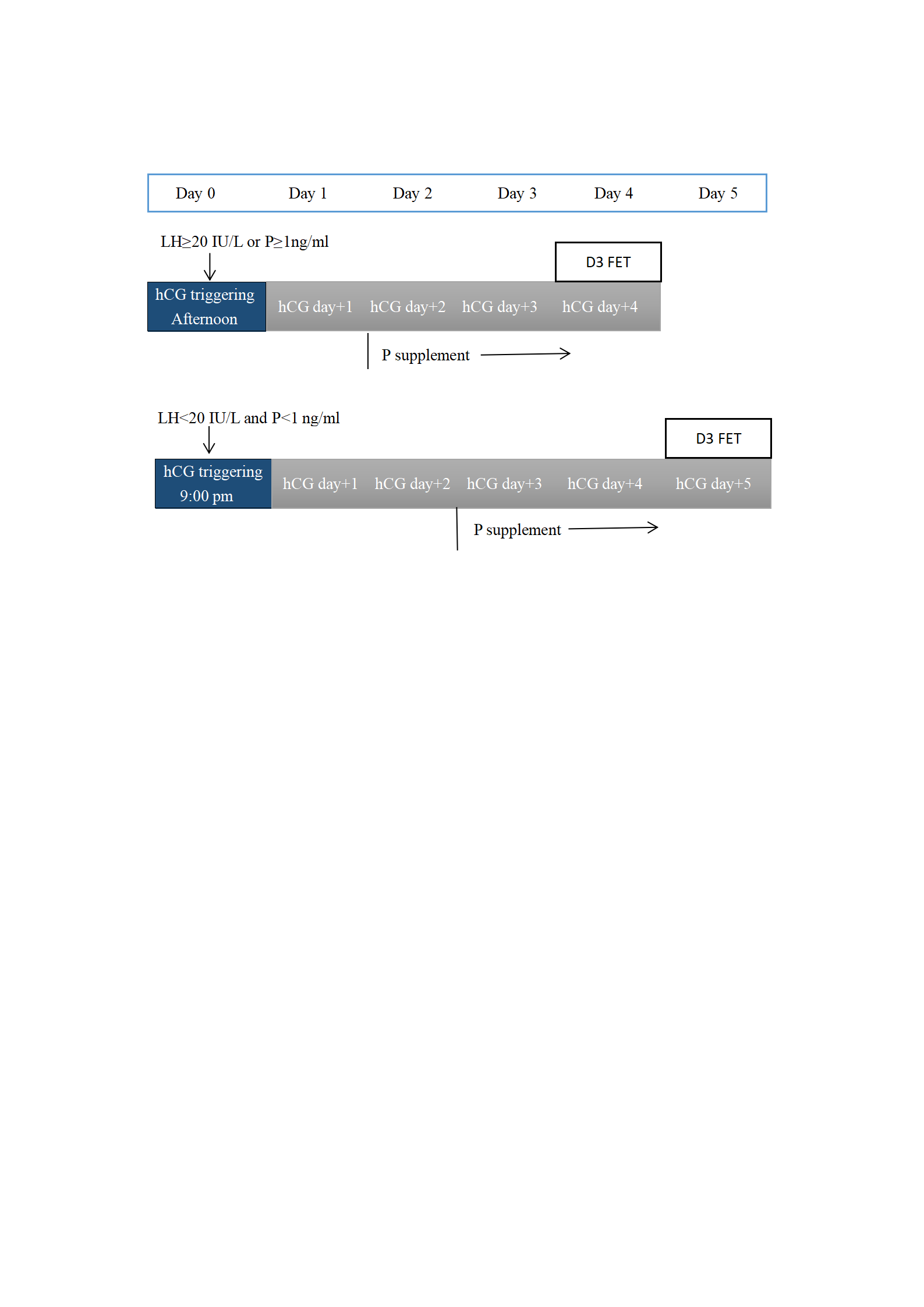
**

Abbreviations: FET = frozen-thawed embryo transfer

**Supplemental Table 1** Comparison of endometrial thickness among natural group and letrozole group (only letrozole group and letrozole +HMG group)

| Endometrial thickness, n (%) | Natural cycle  (*n* = 3958) | Letrozole  (*n* = 1028) | letrozole +HMG  (*n* = 1888) | *P*-value |
| --- | --- | --- | --- | --- |
| <8.2 | 395(10.0) | 112(10.9) | 152(8.1) | 0.080 |
| 8.2-10.5 | 1058(26.7) | 279(27.1) | 502(26.6) |  |
| 10.6-13.6 | 2147(54.2) | 531(51.7) | 1041(55.1) |  |
| ≥13.7 | 358(9.0) | 106(10.3) | 193(10.2) |  |

**Note:** Data are given as *n* (%) for dichotomous variables. All P values were assessed with the use of x^2^ test.

**Supplemental Table 2** Crude and adjusted odds ratios (ORs) of neonatal outcomes between the natural and letrozole groups in the period from 2013 to 2019

|  | Before matching | | | | After matching | | | |
| --- | --- | --- | --- | --- | --- | --- | --- | --- |
|  | Crude OR  (95% CI) | *P*-  value | Adjusted OR  (95% CI) | *P*-  value | Crude OR  (95% CI) | *P*-  value | Adjusted OR  (95% CI) | *P*-  value |
| **Singleton** |  |  |  |  |  |  |  |  |
| Low birth weight (<2500 g) | 1.29(0.81-2.05) | 0.287 | 1.30(0.81-2.08) | 0.279 | 1.51(0.89-2.57) | 0.129 | 1.57(0.92-2.69) | 0.098 |
| Preterm birth (<37 weeks) | 1.06(0.70-1.60) | 0.799 | 1.10(0.72-1.67) | 0.675 | 1.21(0.76-1.93) | 0.419 | 1.30(0.81-2.07) | 0.276 |
| Major congenital anomalies | 1.35(0.69-2.63) | 0.379 | 1.19(0.61-2.36) | 0.609 | 1.22(0.58-2.57) | 0.608 | 1.18(0.56-2.51) | 0.661 |
| **Twins** |  |  |  |  |  |  |  |  |
| Low birth weight (<2500 g) | 1.04(0.84-1.27) | 0.740 | 1.09(0.85-1.39) | 0.505 | 1.02(0.81-1.28) | 0.873 | 1.04(0.82-1.32) | 0.750 |
| Preterm birth (<37 weeks) | 1.23(0.99-1.52) | 0.058 | 1.21(0.94-1.57) | 0.137 | 1.10(0.87-1.39) | 0.437 | 1.09(0.86-1.39) | 0.473 |
| Major congenital anomalies | 1.45(0.70-3.02) | 0.323 | 1.76(0.82-3.79) | 0.148 | 0.72(0.36-1.45) | 0.360 | 0.81(0.40-1.64) | 0.554 |

**Note:** Analyses were adjusted for age, body mass index, duration of infertility, cause of infertility, duration of cryopreservation, number of transferrable embryos, embryo quality at transfer, endometrium thickness.

Abbreviations: OR = odds ratio, CI = confidence interval
